# Manual of Obstetrics

**Published:** 1910-12

**Authors:** 


**The Medical Record Visiting List or Physician's Diary for 1911.** New revised edition. New York: William Wood & Company, Medical Publishers. 1910.

In the December issue of this magazine for 1909 we published a review notice of this invaluable visiting memorandum book, and we are glad to receive it in season to publish this notice in a corresponding number of the JOURNAL this year. It is important that physicians should be supplied with their visiting lists for next year before 1911 sets in. In the 60 patients a week list before us, besides the space set apart for patients, we find a vast amount of valuable material useful in the sick room or at the bedside, such as a two years' calendar; estimation of the probable duration of pregnancy; approximate equivalents of temperature, weight, capacity, measure, and the like; maximum adult dose by the mouth in apothecaries' and decimal measures; drops in a fluid dram; solutions for subcutaneous injection; solutions in water for atomisation and inhalation; miscellaneous facts, emergencies, surgical antisepsis, disinfection, dentition, and table of signs; visiting list with special memoranda; consultation practice; obstetric engagements, and record of obstetrical practice; record of vaccination; register of deaths, nurses' addresses, addresses of patients and others, cash account, as well as other data and hints of value.

The prices of regular lists are, for sixty patients a week, with or without dates, handsomely selected red or black morocco binding, $1.50; for thirty patients a week with or without dates, same style, $1.25. Orders should be placed with promptitude, lest the edition become exhausted before the end of the present month.

---

**Manual of Obstetrics.** By **A. F. A. King**, A.M., M.D., Professor of Obstetrics in the Medical Department of the George Washington University, Washington, D. C. Eleventh edition. 12 mo. pp. 733. Illustrated. Philadelphia and London: Lea & Febiger. 1910. (Cloth, $2.75.)

The description of the management of normal labor, as well as the treatment of emergencies which arise in the practice of obstetrics, has been made as nearly complete as possible, no detail great or small being overlooked. In this the eleventh edition of his work, King retains all the valuable features of former revisions and has added new material to bring it down to the present moment. Lucidity of statement and avoidance of repetition mark its pages to a high degree. The brevity with which some subjects are discussed does not sacrifice completeness. As a manual for the student and the busy practitioner it will continue to enjoy its well-merited reputation. J. A. R.

